# Computational method for estimating progression saturation of analog series†

**DOI:** 10.1039/c7ra13748f

**Published:** 2018-01-31

**Authors:** Ryo Kunimoto, Tomoyuki Miyao, Jürgen Bajorath

**Affiliations:** Department of Life Science Informatics, B-IT, LIMES Program Unit Chemical Biology and Medicinal Chemistry, Rheinische Friedrich-Wilhelms-Universität Dahlmannstr. 2 D-53113 Bonn Germany bajorath@bit.uni-bonn.de +49-228-2699-341 +49-228-2699-306

## Abstract

In lead optimization, it is difficult to estimate when an analog series might be saturated and synthesis of additional compounds would be unlikely to yield further progress. Rather than terminating a series, one often continues to generate analogs hoping to reach the final optimization goal, even if obstacles that are faced ultimately prove to be unsurmountable. Clearly, methodologies to better understand series progression and saturation are highly desirable. However, only a few approaches are currently available to monitor series progression and aid in decision making. Herein, we introduce a new computational method to assess progression saturation of an analog series by relating the properties of existing compounds to those of synthetic candidates and comparing their distributions in chemical space. The neighborhoods of analogs are analyzed and the distance relationships between existing and candidate compounds quantified. An intuitive dual scoring scheme makes it possible to characterize analog series and their degree of progression saturation.

## Abstract 2

Chemical space view of an analog series. Shown are inactive (red) and active (blue) analogs together with virtual candidate compounds (green) available to expand the series. Chemical neighborhoods of analogs are depicted in gray.

## Graphical abstract

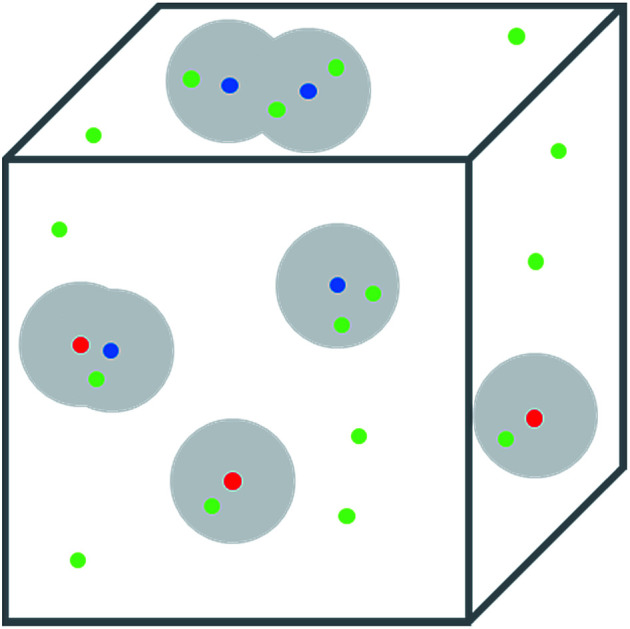

## Introduction

Key challenges governing the practice of medicinal chemistry can be expressed in simple terms. First and foremost, there is the pivotal question that rules the daily life of medicinal chemists in hit-to-lead and lead optimization campaigns: “Which compound to make next?” This question focuses on structure–activity relationship (SAR) progression aiming at the ultimate success of lead optimization.^1^ Equally relevant are the questions “how many more compounds should we make?” or, in other words, “when should we stop?” Here, the issues of progression saturation and SAR redundancy take center stage: one would like to better understand if further SAR progression can be expected by making more compounds or if analog space has been saturated and it is time to discontinue synthetic efforts. Such decisions are generally difficult to make. First and foremost, the ability to predict SAR progression is only limited at present. Moreover, from a behavioral standpoint, it is often a difficult decision to discontinue medicinal chemistry projects that have been advanced with hard and diligent work over time, yet do not quite reach the desired goal. In such situations, it is – from an investigator's perspective – typically easier to continue optimization efforts and hope for a breakthrough with the next compound(s) than to terminate a project. At the same time, it is of critical importance to recognize the presence of insurmountable obstacles during optimization and avoid a prolonged and increasingly costly waste of time and efforts. This is a major problem in the practice of medicinal chemistry. Hence, one would very much like to rationalize such decision processes and determine criteria that can be applied to reliably predict SAR progression and analogs that should best be made. While the latter task represents the grand challenge of quantitative SAR (QSAR) analysis methods,^2^ the former can also be addressed by determining when a sufficiently high degree of progression saturation of an analog series has been reached. However, making decisions about new compounds and judging the odds of an optimization project are far from being routine and continue to be largely driven by chemical experience, intuition, or ingenuity, rather than by methodological concepts that can be translated into predictive methods.

Going beyond QSAR analysis with its long history in medicinal chemistry, only few computational approaches have been introduced to systematically predict new analogs and explore SAR progression or chemical saturation. For example, given the multi-parametric nature of lead optimization and the need to balance several properties in addition to potency such as solubility, stability, or bioavailability, multi-objective optimization using probability or desirability functions has been applied to assist in compound design.^3^ In addition, attrition analysis has been introduced to prioritize candidate compounds on the basis of optimization criteria defined as ranges of different numerical properties.^4^ Attrition curves have been generated to report the number of compounds meeting varying numbers of criteria during optimization.^4^ These approaches require the initial definition of desirable properties, rendering them partly subjective. Furthermore, activity landscape models have been used to visualize SAR information associated with evolving compound sets and qualitatively evaluate SAR progression.^5^ Similarly, ensembles of SAR matrices have been generated and scored using SAR analysis functions to monitor SAR characteristics of compound series over time.^6^ In scatter plots, defined shifts of matrix ensembles towards SAR discontinuity or continuity have revealed SAR progression or redundancy on a time course.^6^ These approaches are diagnostic in nature and do not aid in compound design. Following another route, a statistical methodology has been introduced to quantify optimization risk on the basis of multiple numerical properties.^7^ Applying this framework, positive SAR progression minimizes the statistical risk associated with optimization and test compounds making significant contributions to risk minimization can be identified.^7^

Given the limited number of computational concepts and methods that are currently available to assess SAR progression and chemical redundancy, there continues to be much room for new developments. Herein, we introduce a new computational approach to quantify chemical progression saturation of evolving analog series that compares existing and virtual candidate compounds in chemical space, quantifies global and local relationships between compounds, and characterizes neighborhoods of existing analogs.

## Methods and materials

### Chemical space representation

For all calculations, a constant chemical reference space was generated on the basis of seven numerical descriptors accounting for molecular properties that are known to strongly influence biological activities of compounds. These descriptors included molecular weight, the number of hydrogen bond donor and acceptor atoms, respectively, the number of rotatable bonds, the logarithm of the octanol/water partition coefficient (log *P*), aqueous solubility, and topological polar surface area. Hence, these descriptors account for molecular size, hydrogen bonding capacity, flexibility, hydrophobicity, solubility, and polar surface area. Descriptors were calculated with the Molecular Operating Environment (MOE)^8^ and scaled to zero mean and unit variance. To assess compound relationships in scaled descriptor space, Euclidian distances were calculated. Principal component analysis (PCA) of descriptor space was carried out using Python scripts with the aid of Scikit-Learn.^9^ We note that for the methodology reported herein, a variety of chemical space representations can be employed, dependent on given preferences or project requirements. For our proof-of-concept study, a chemically intuitive reference space of limited dimensionality was chosen that represented molecular features of high relevance for biological activity. It is emphasized that the methodology presented herein does not depend on chosen descriptor sets.

### Analog series

For the analysis of progression saturation, analog series with single-site substitutions were extracted from the PubChem BioAssay database^10^ using a computational method^11^ based upon the matched molecular pair (MMP) concept.^12^ An MMP is defined as a pair of compounds that are only distinguished by a chemical change at a single site.^12^ MMPs were systematically generated by single-cut fragmentation of exocyclic single bonds^13^ on the basis of retrosynthetic rules,^14^ yielding RECAP-MMPs.^15^ From each analog series, the RECAP-MMP core was isolated. For each series, the analog with highest ligand efficiency (LE)^16^ was determined and defined as the “lead compound”.
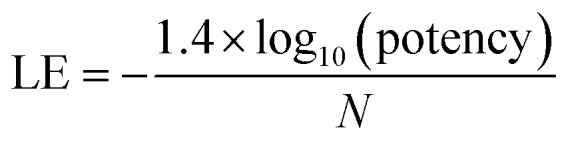
here, *N* is the number of non-hydrogen atoms and potency is accounted for on the basis of IC_50_ values.

We note that analog series were extracted from PubChem BioAssays to include both active and inactive compounds in the analysis. Qualifying series were required to consist of at least 30 analogs tested in the same assay and contain at least three active compounds.

We selected 80 analog series from 25 biochemical assays covering 23 different targets and comprising a total of 1618 compounds. Table S1 of the ESI† reports the composition of all analog series, their targets, and the assays the series originated from. The analog series were active against a variety of targets including, for example, thyroid hormone receptor, ATPases, glucagon like peptide 1 receptor, or serine/threonine protein kinase PLK1. All compound data sets are freely available upon request.

### Virtual compounds

Virtual candidate compounds were generated by systematic recombination of RECAP-MMP core structures extracted from PubChem analog series with substituent fragments obtained from active compounds taken from ChEMBL.^17^ Therefore, from ChEMBL release 23, compounds with direct interactions (type “D”) with human targets at the highest assay confidence level (confidence score 9) were selected. Only equilibrium constants (*K*_i_ values) and IC_50_ values were considered as potency measurements. Approximate measurements (*e.g.*, “>” or “∼”) were discarded. On the basis of these selection criteria, 244 625 compounds were obtained and subjected to the same RECAP-MMP fragmentation procedure as PubChem compounds. On the basis of systematic fragmentation, a total of 13 203 unique substituent fragments from ChEMBL compounds were identified that were not present in PubChem analogs. These fragments were then recombined with the RECAP-MMP core of each analog series, yielding a total of novel 13 203 virtual analogs per series. By design, these virtual compounds combined core structures from active screening compounds and substituents of active compounds from the medicinal chemistry literature and were used as candidates for series progression. Virtual compounds were generated with Python scripts making use of the OpenEye toolkit.^18^

### Progression saturation scores

Assayed analogs were mapped into chemical reference space on the basis of their calculated descriptor coordinates. Neighborhoods of assayed analogs in chemical reference space were defined on the basis of distance relationships to nearest virtual or existing neighbors. The heuristic distance criteria specified below can be readily adjusted depending on chosen chemical space representations and/or compound series under investigation.

**Fig. 1:**
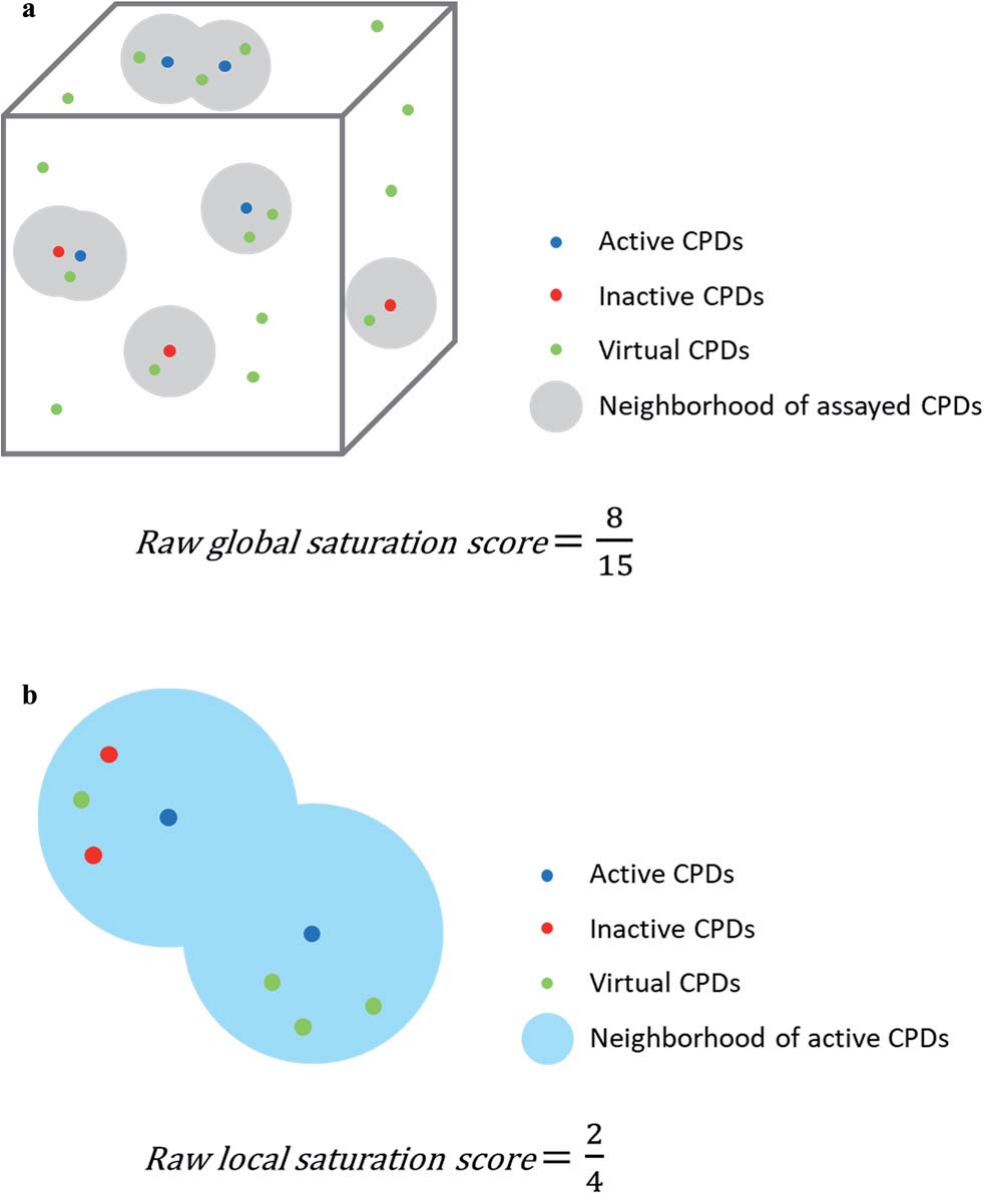
Score concept. In (a) and (b), the calculation of the raw global and local saturation scores is schematically illustrated, respectively. The box represents chemical space populated with a single analog series including virtual candidate compounds.

The raw global saturation score was defined as the ratio of the number of virtual candidate compounds falling into the neighborhoods of assayed (active or inactive) analogs relative to the total number of virtual compounds:
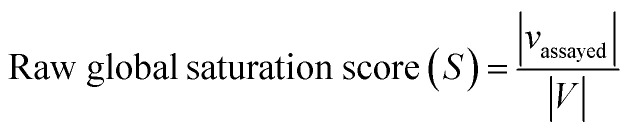
here, *S* represents the set of assayed analogs, *V* the set of virtual compounds, and *ν*_assayed_ the set of virtual compounds falling into neighborhoods of assayed analogs. In this case, the neighborhood radius for each analog was set to the median value of the distribution of Euclidian distances between each virtual compound and the top 1% nearest virtual neighbors. Thus, the neighborhood radius around assayed analogs was determined by the distance distribution between virtual neighbors ensuring that only closely related virtual candidates would map to the same neighborhood. As defined, the raw global saturation score, in the following also referred to as global score, quantifies the bilateral chemical space coverage of existing analogs and virtual compounds, *i.e.*, the larger the score is, the more virtual candidates fall into the neighborhoods of existing compounds, indicating extensive coverage by both. For estimating progression saturation of series, the global score primarily serves as a measure for chemical space coverage of existing analogs. The calculation of the raw global score is illustrated in Fig. 1a. The raw scores were converted into conventional *z*-scores on the basis of the mean and standard deviation of the score distribution of all analog series.

The raw local saturation score was defined as the ratio of the number of active analogs relative to the number of virtual compounds falling into the neighborhoods of active analogs:
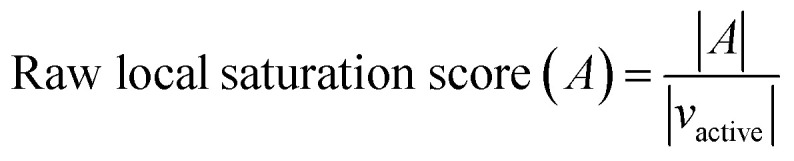
here, *A* is a set of active compounds and *ν*_active_ the set of virtual compounds falling into the neighborhoods of active compounds. In this case, the radius of the neighborhood of each active analog was set to the median value of the pairwise distances between active analogs. Accordingly, the size of the neighborhood corresponded to typical distances between active compounds and was hence activity-relevant. As defined, the raw local saturation score, in the following also referred to as local score, is a measure for the distribution of virtual candidates around existing active analogs, *i.e.* the larger the score is, the less populated neighborhoods of active analogs are with virtual candidates. The calculation of the raw local score is illustrated in Fig. 1b. Raw local scores were also converted into *z*-scores after logarithmic transformation.

So-defined global and local scores are complementary in nature and make it possible to monitor the progression of an analog series and quantify its saturation by assessing the chemical space coverage of analogs together with the saturation of “active neighborhoods”.

## Results and discussion

### Methodological concept

The assessment of progression saturation of analog series is based upon two criteria. First, distributions of existing analogs and virtual candidate compounds are globally compared. Second, virtual neighbors of active analogs are identified. In both instances, a chemical neighborhood concept^19–21^ is applied, but in different ways. First, the more virtual compounds fall into chemical neighborhoods of analogs, regardless of their activity, the larger the chemical space coverage of an analog series is. This criterion accounts for chemical diversity of analogs sharing a given core structure. The degree of coverage represents a progression saturation criterion for analog series, which is accounted for by the global saturation score, as discussed above (Fig. 1a). Here, it is important that chemical neighborhoods of analogs are defined by near neighbor distances between virtual compounds in order to ensure a consistent comparison of compound distributions. Second, an alternative definition of neighborhoods of active analogs is applied that is based upon the mean distance between active compounds and leads to an assessment of saturation from a different perspective, accounted for by the local saturation score (Fig. 1b). In this case, likelihood of activity of candidate compounds in neighborhoods of existing analogs^19^ is the key criterion. Accordingly, low coverage of “active neighborhoods” by virtual analogs is indicative of progression saturation. Thus, there are only few candidate compounds left that are likely to be active. By contrast, as long as many virtual candidates fall into active neighborhoods, corresponding to a low local score, the progression of the series is not yet saturated. Therefore, it is important to define neighborhoods of active compounds using activity-relevant radii, as discussed above. Furthermore, because saturation assessment depends on prospective candidate compounds (*i.e.*, next analogs to be made), it is also important to generate virtual compounds that are suitable for series progression. These candidates must contain the core structure of a series and activity-relevant substituents, as also discussed above. Therefore, we have implemented a new analog generation approach different from conventional combinatorial analog enumeration that specifically meets these requirements. Combining the different saturation criteria, *i.e.*, assessing compound coverage as well as the population of active neighborhoods, makes it possible to estimate the chemical progression saturation of analog series in a differentiated manner, as discussed in the following.

It is noted that any similarity- or distance-based chemical neighborhood and nearest neighbor approach^19,20^ is representation-dependent.^20,21^ Hence, this dependence is general and not related to the specifics of our methodology. As discussed above, progression saturation analysis is applicable to any chosen chemical reference space, without restrictions, and does not depend on a particular representation (or similarity measure).

Furthermore, it is emphasized that the new methodology primarily focuses on assessing chemical criteria associated with saturation, rather than SAR discontinuity, which requires taking compound potency variations into account. SAR progression is assessed by analyzing if newly generated compounds further increase SAR discontinuity or if they retain similar potency, despite chemical modifications. SAR discontinuity assessment represents another form of saturation analysis. It can also be rationalized as an estimation of the likelihood that the generation of additional analogs will yield activity cliffs. By contrast, the method introduced herein employs criteria related to the chemical diversity of analogs and addresses the question how saturated analog space might be and how likely it would be to identify new active analogs.

In the following, the results of our proof-of-concept investigations are presented and discussed.

### Score distributions

**Fig. 2:**
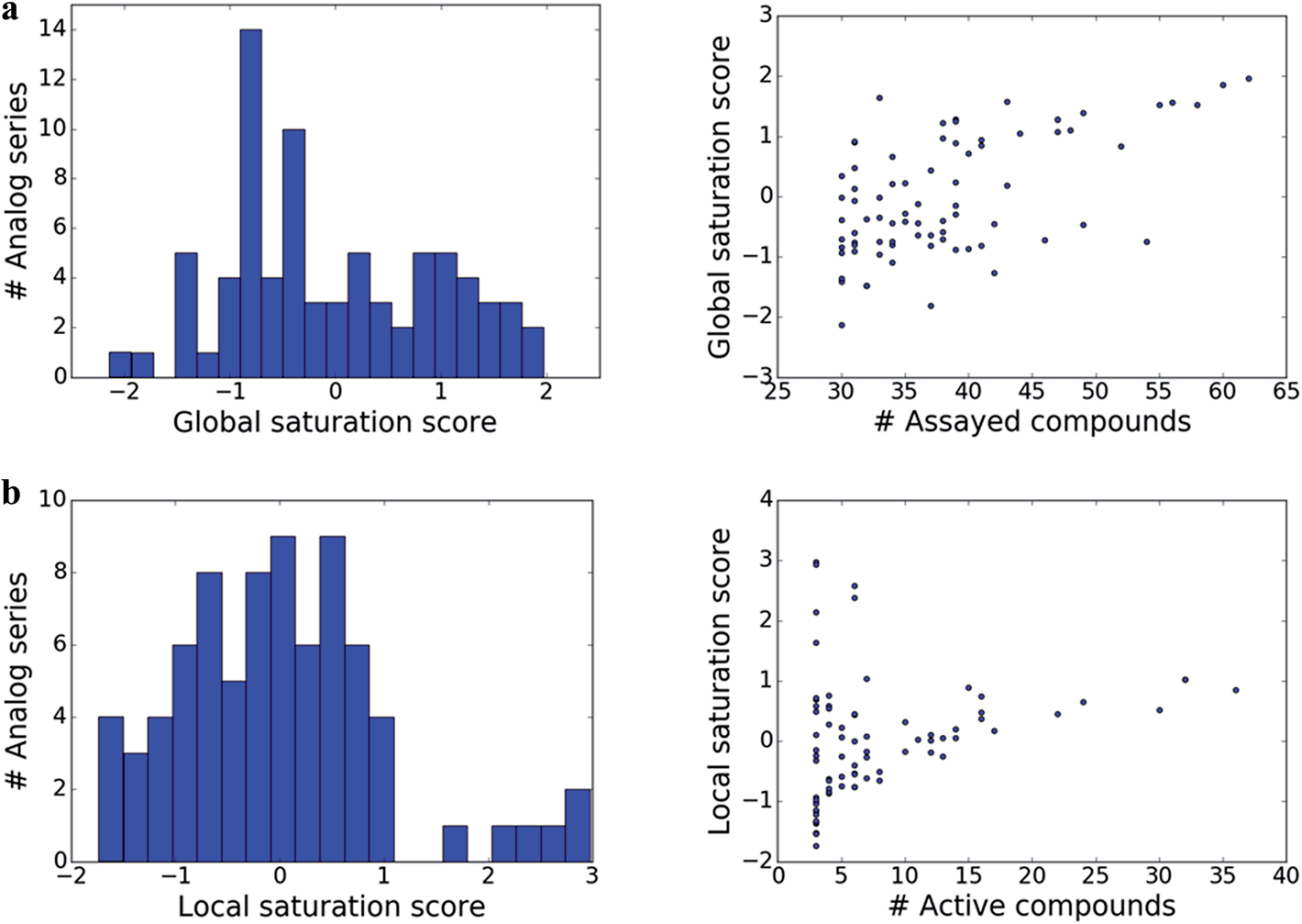
Distribution of scores. Histograms (left) report the distribution of the (a) global and (b) local score over analog series. In addition, scatter plots (right) relate (a) global scores to the number of assayed (active or inactive) analogs per series and (b) local scores to the number of active analogs.

The distributions of global and local scores for the 80 analog series are shown in Fig. 2a (left) and Fig. 2b (left), respectively. Raw scores were transformed into conventional *z*-scores. Both global and local scores were widely distributed covering the range of [−2, 2] and [−2, 3], respectively. Furthermore, Fig. 2 also shows that there was no significant correlation between the magnitudes of global and local scores and the total number of assayed and active compounds per series, respectively (with correlation coefficients of 0.57 and 0.28). Only the largest analog series displayed a tendency to produce high global scores (Fig. 2a, right), as one might expect. In addition, some but not all series containing small numbers of active compounds produced high local scores (Fig. 2b, right), as one would also anticipate. Taken together, the distributions of global and local scores indicated that these scores were capable of differentiating between analog series of varying composition.

### Characteristic score combinations

**Fig. 3:**
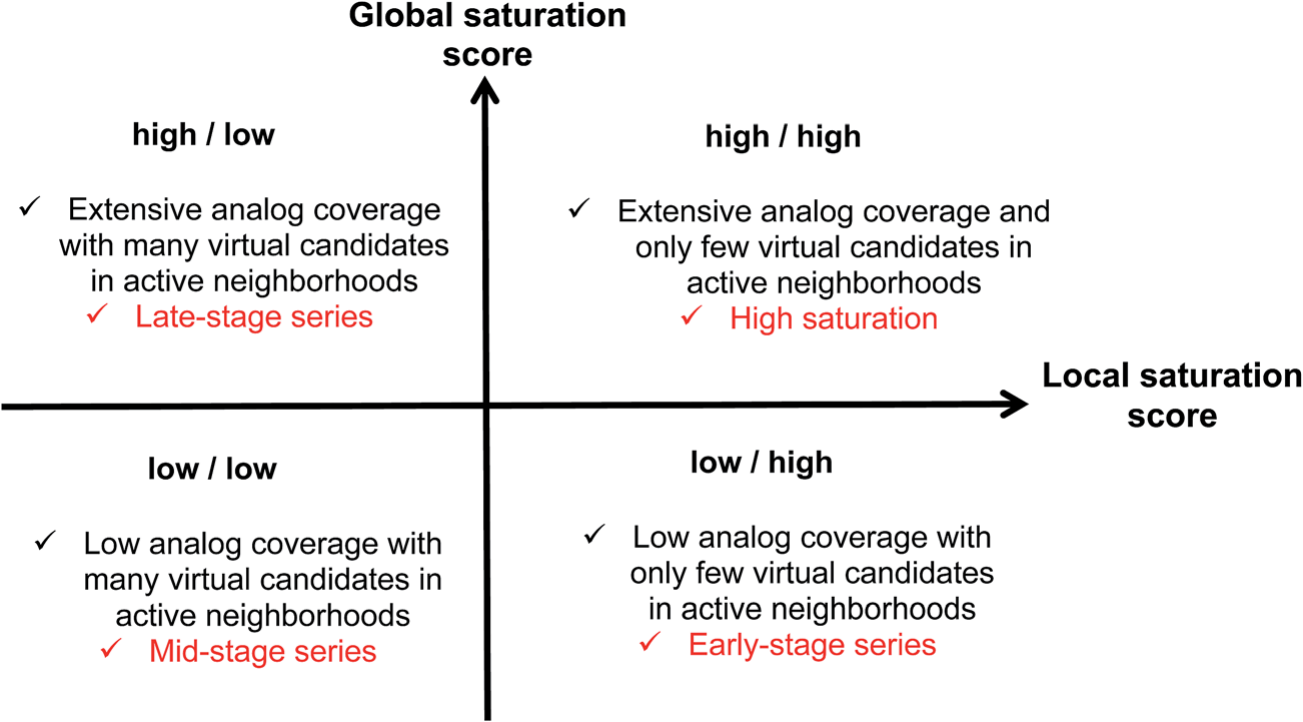
Score combinations. Combinations of global and local saturation scores of different magnitude (high, low) are rationalized. “Analog coverage” refers to the coverage of chemical space with existing analogs.

Combining the global and local score yielded a dual scoring scheme that made it possible to distinguish characteristic score combinations and assign analog series to different saturation categories, as summarized in Fig. 3. These categories reflect different stages for compound synthesis. For example, the combination of low global and high local scores (lower right in Fig. 3) is indicative of series with overall low compound coverage and only few virtual candidates mapping to active neighborhoods. Such series would be characteristic of early-stage optimization projects when only little SAR information is available. Hence, some exploratory chemistry efforts would be required. In addition, the low/low combination of global and local scores (lower left) identifies series with still limited compound coverage but many virtual candidates in neighborhoods of active compounds. Thus, in such cases, “close-in analogs” of active compounds are beginning to be identified. Accordingly, such analog series are best rationalized as intermediate or mid-stage series. We note that an exception would be the situation when only very few analogs but many virtual compounds were available, which would also favor a low/low score combination. Furthermore, the high/low score combination (upper left) is indicative of series with extensive compound coverage, yet still many remaining virtual candidates in active neighborhoods. This would be characteristic of late-stage series approaching saturation. Finally, the high/high score combination (upper right) reveals series with extensive compound coverage and only few remaining virtual candidates in active neighborhoods, which corresponds to a high degree of saturation. Therefore, the high/high score combination provides a saturation alert for series under investigation.

Considering the distributions of global and local scores for our ensemble of analog series (Fig. 2), high global and local scores corresponded to *z*-scores ≥1 (*i.e.*, one sigma above the mean of fitted pseudo-normal distributions). These threshold values can be used as a diagnostic for practical applications. Taken together, the score combinations in Fig. 3 characterize a spectrum of series at different stages of chemical optimization.

### Score comparison

**Fig. 4:**
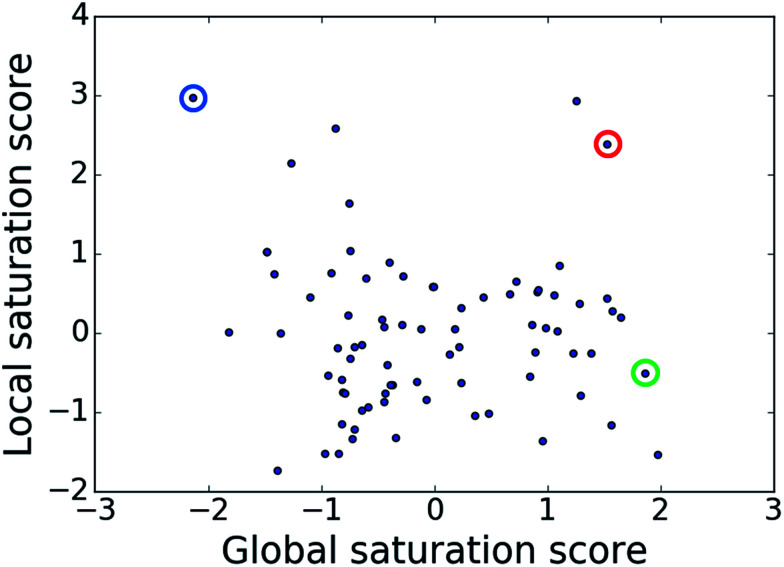
Score comparison. For all analog series, global and local saturation scores are compared. Colored circles mark three exemplary series with high/high (red), high/low (green), and low/high (blue) global/local score combinations, respectively. For these series, further details are provided in Table 1.

The comparison of global and local scores for the 80 analog series is shown in Fig. 4, providing an alternative view of the score distributions. Three exemplary series with distinctive high/high (red), high/low (green), and low/high (blue) scores are highlighted. The core structures, composition, and scores of these series are reported in Table 1. Strikingly, the series with high/high and high/low scores contained the same core structure and were active against unrelated targets. As expected, given the conserved set of virtual candidate compounds and overlapping yet distinct sets of assayed analogs, the global scores were comparable. By contrast, the local scores differed greatly, reflecting distinct distributions of active analogs and different SAR characteristics. The series with activity against RAR-related orphan receptor gamma was highly saturated, whereas the corresponding series with activity against lysine-specific demethylase 4A still contained many virtual candidates in neighborhoods of active analogs that might be considered for synthesis. This comparison also illustrates that different target-specific SAR characteristics of corresponding series can be detected by global/local scoring.

**Table 1:** Analog series and scores^a^

Global/local	Core structure	AID target	# active CPDs	# assayed CPDs	Global saturation score	Local saturation score
High/high	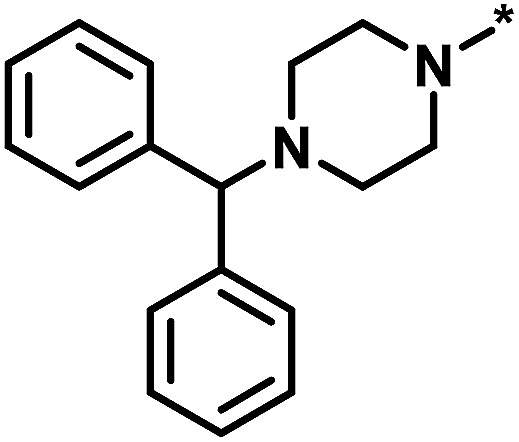	2551, RAR-related orphan receptor gamma	6	58	1.52	2.39
High/low	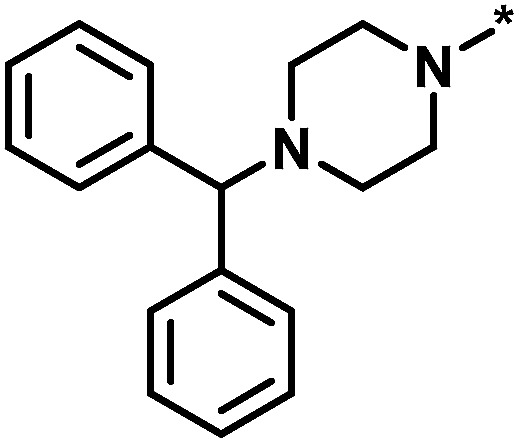	504339, lysine-specific demethylase 4A	8	60	1.86	−0.51
Low/high	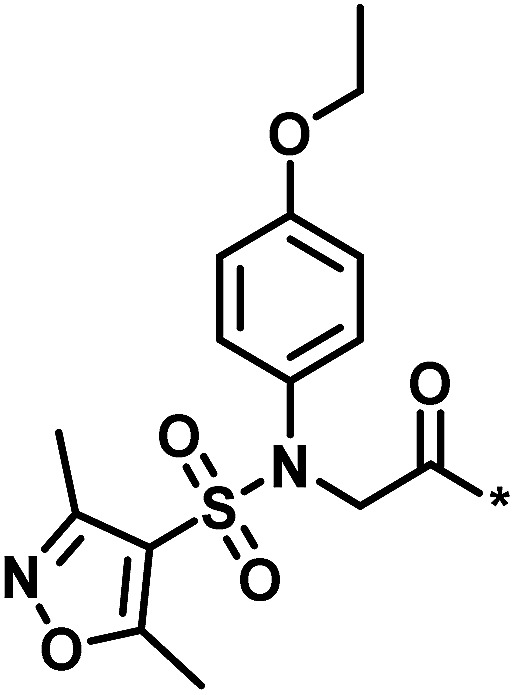	504444, erythroid 2-like nuclear factor	3	30	−2.14	2.97

a The table reports global and local SAR saturation scores for the three analog series highlighted in Fig. 4. The corresponding score combinations are given and the core structures of the series are shown. In addition, the PubChem assay ID (AID) and target of each series are provided and the number of active compounds (CPDs) and total number of compounds (assayed CPDs) per series are reported.

### Chemical space projections

**Fig. 5:**
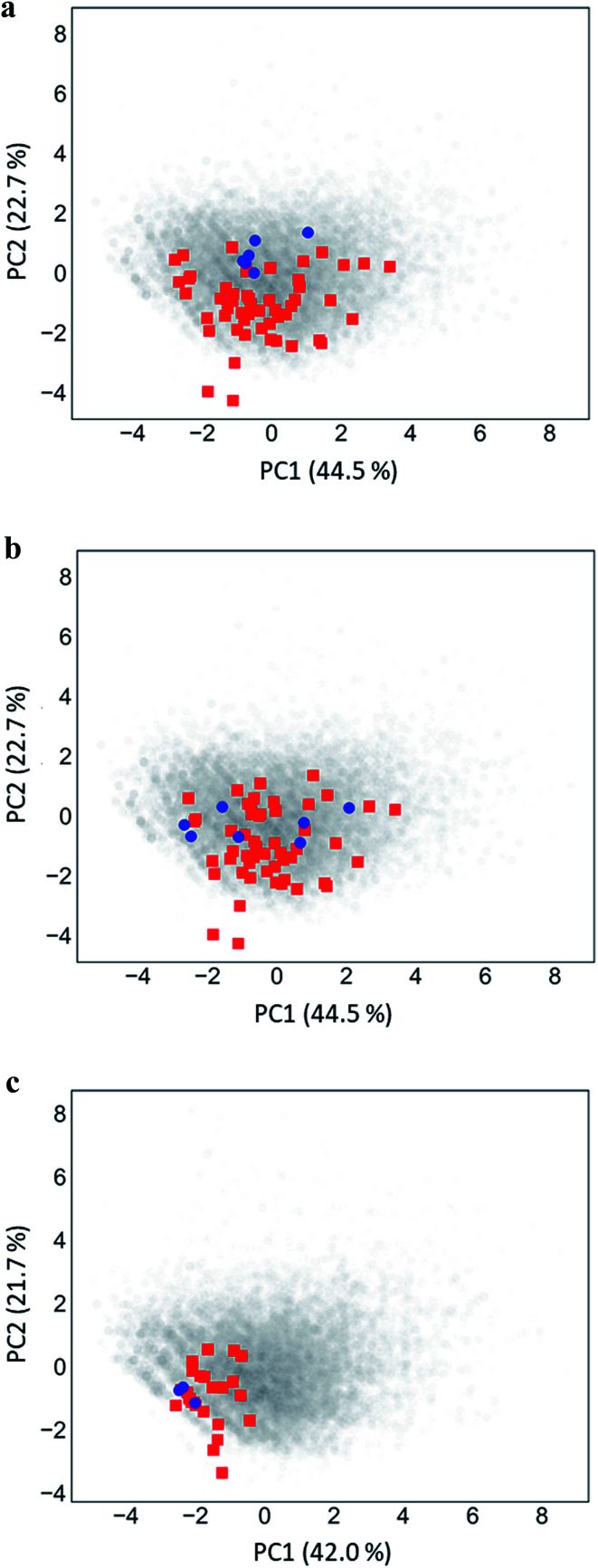
Principal component analysis. Shown are principal component plots for the exemplary analog series in Fig. 4 with (a) high/high, (b) high/low, and (c) low/high global/local score combination. The plots represent two-dimensional projections of descriptor space for series (blue, active analogs; red, inactive) including all virtual compounds (gray). Percentages report the contributions of the first (PC1) and second (PC2) principal component to data variance.

Fig. 5 shows PC plots for the three analog series in Table 1 that reveal differences in compound distributions leading to distinct score combinations. Fig. 5a and b show chemical reference space projections for the two series with identical core structure and high/high and high/low scores, respectively. The plots confirm extensive chemical space coverage of existing analogs in both cases, consistent with high global scores, and highlight a key difference between the two series that led to local scores of different magnitude: while inactive compounds (red) were very similarly distributed in both cases, most active compounds in Fig. 5a (high/high scores) were located close to each other, whereas active compounds in Fig. 5b (high/low) were widely distributed. Hence, in the latter case, neighborhoods of active compounds were larger and contained more virtual candidates. Furthermore, Fig. 5c shows the projection for a chemically distinct analog series with activity against the erythroid 2-like nuclear factor that was characterized by low/high scores. In this case, there was only limited analog coverage of chemical space and small overlap between existing analogs and virtual candidates. In addition, there were only three active analogs that mapped to similar positions. Taken together, these observations illustrated the early-stage character of this series, consistent with its low/high score combination.

**Fig. 6:**
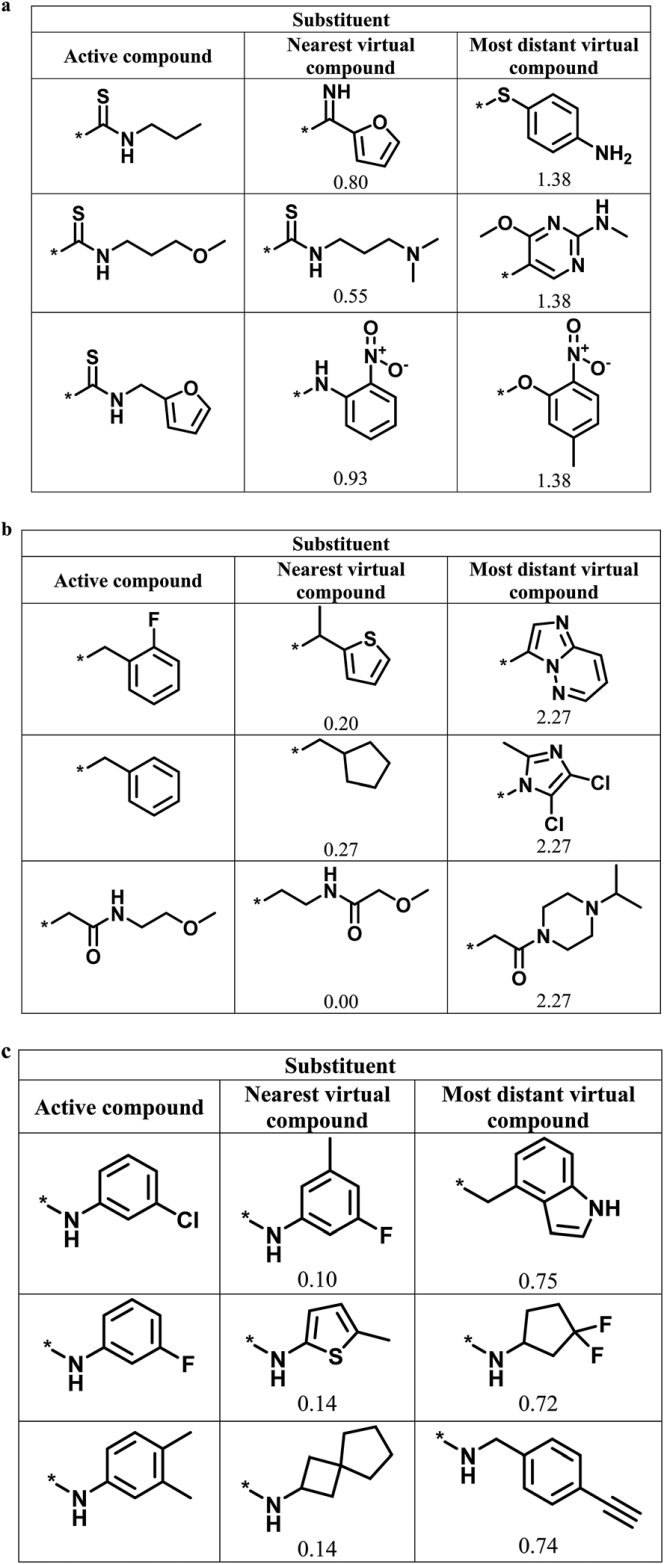
Active analogs and virtual candidates. For the series in Fig. 4 with (a) high/high, (b) high/low, and (c) low/high global/local score combination, substituents of representative active analogs are shown together with substituents of the nearest virtual neighbor and the most distant virtual compound within neighborhoods of active analogs in chemical space. For virtual compounds, Euclidian distances from active analogs are reported.

Fig. 6 shows representative active analogs and virtual candidates for the three series with different score combinations, which reveal similarities and chemical differences between these compounds, depending on their distance relationships.

### Alternative series

Table 2 reports the composition and scores for four series of analogs that were active against the same target, tyrosyl-DNA phosphodiesterase 1, which contained distinct core structures. These series were characterized by different score combinations. At a first glance, series 2, which contained the largest number of active analogs, might be considered the most advanced series. However, if global and local scores were taken into consideration, a different picture emerged. Series 1 and even more so series 3 had high global and low local scores, indicating the availability of virtual candidates in active neighborhoods in the presence of extensive compound coverage. In contrast to series 1, there were no active analogs in the neighborhood of the lead compound of series 3, indicating an opportunity for further chemical exploration, consistent with the much lower local score of series 3 compared to 1. Furthermore, series 2 had a low global and comparably high local score, reflecting much lower analog coverage than detected for series 1 and 3 and the presence of fewer virtual candidates in active neighborhoods. By contrast, series 4 was characterized by low/low scores, consistent with the presence of more virtual candidates in active neighborhoods. Thus, on the basis of these score characteristics, chemical exploration of series 1 and 3 was more advanced than of series 2 and 4. However, despite its early-stage character, series 2 already contained more active analogs than the other series. Accordingly, one would assign priority to this series, given its lower degree of saturation and high potential for further chemical exploration, expecting to identify more active compounds. These comparisons show how alternative series might be prioritized on the basis of different score combinations.

**Table 2:** Scores for multiple analog series active against the same target^a^

No.	Core structure	# active CPDs	# assayed CPDs	Global score	Local score	Lead substituent	Lead # active NBs
1	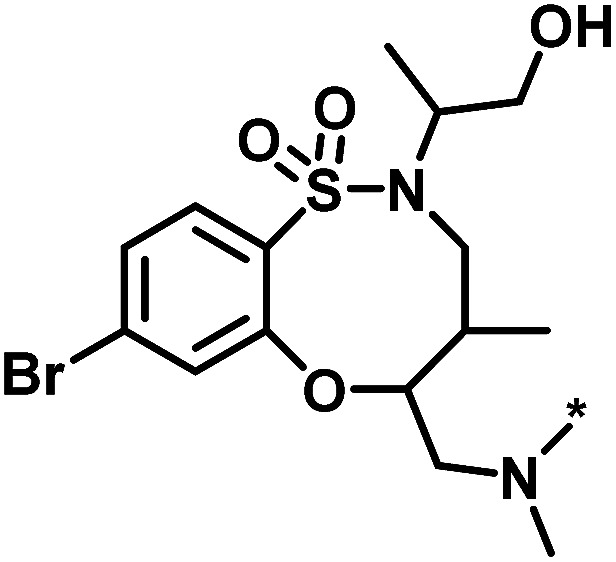	11	47	1.08	0.02	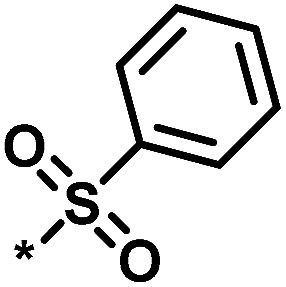	7
2	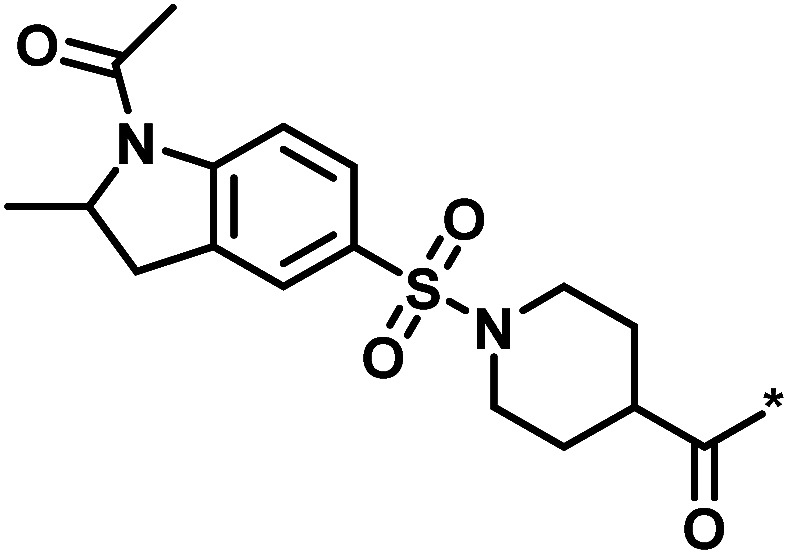	16	30	−1.42	0.75	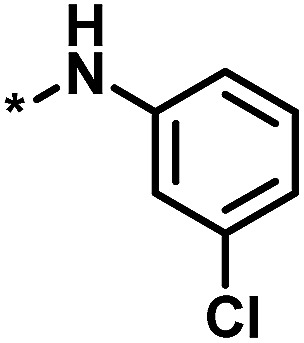	11
3	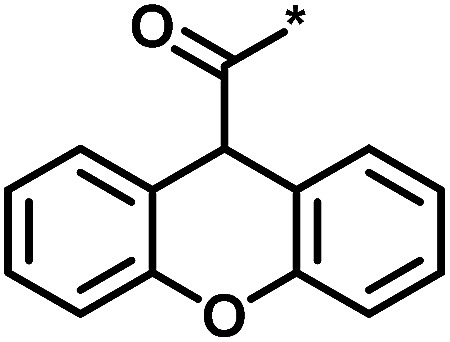	4	39	1.29	−0.79	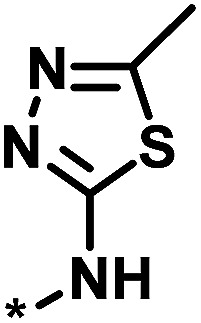	0
4	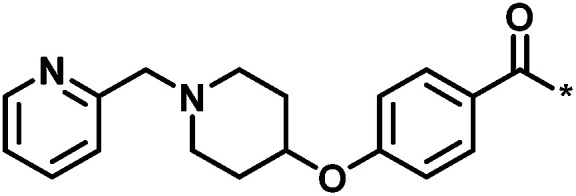	3	33	−0.96	−1.52	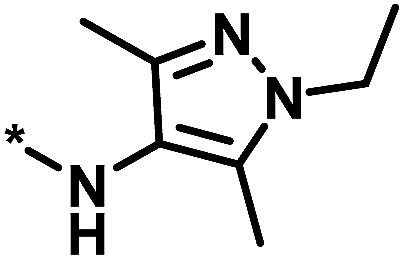	1

a The table reports global and local scores for four exemplary analog series active against tyrosyl-DNA phosphodiesterase 1 (AID 686979). For each series, the core structure is shown and the number of all assayed and active compounds (CPDs) is reported. In addition, for the lead compound (lead) of each series, the substituent is shown and the number of active neighbors (NBs) is reported (*i.e.*, active analogs falling into the neighborhood of the lead compound).

## Conclusions

The assessment of SAR progression and chemical saturation of analog series presents substantial challenges in medicinal chemistry. The question when a sufficiently large number of analogs is available to judge the potential of a series to reach its final optimization goal is of high practical relevance. On the one hand, one would avoid giving up too early on a candidate series, on the other, continuation of optimization efforts might lead to a significant waste of time and resources, without reaching the final goal. Clearly, any approaches to quantitatively assess – and ultimately predict – series progression and/or saturation are of high interest for medicinal chemistry. However, so far only few computational concepts have been put forward to rationalize series progression and – to our knowledge – no approach is currently available to estimate the saturation of series. Herein, we have reported a first attempt in this direction, introducing a computational method to compare the distributions of existing analogs and virtual candidate compounds for synthesis and analyze chemical neighborhoods of analogs. By design, the approach avoids extensive calculations and operates in intuitive chemical feature spaces. It requires, however, the generation of sets of virtual candidates and is hence not applicable if only one new candidate is considered at a given point in time. A key feature of our methodology is that it provides a simple scoring scheme. As we have demonstrated for a variety of model series extracted from assays, the calculation of global and local saturation scores yields characteristic score combinations that make it possible to differentiate between alternative analog series, identify series with a high degree of saturation, and prioritize others having potential for further chemical exploration in the search for new active compounds. These simple scores can be readily calculated to provide practical guidance for saturation analysis of evolving analog series.

## Conflicts of interest

There are no conflicts of interest to declare.

## Supplementary Material

RA-008-C7RA13748F-s001
