# Supplementary material for: Computational method for estimating progression saturation of analog series
Source: RSC Adv. 2018 Jan 31;8(10):5484–92. doi: 10.1039/c7ra13748f (PMC9078142; doi:10.1039/c7ra13748f)
Supplement: RA-008-C7RA13748F-s001 [file RA-008-C7RA13748F-s001.pdf]

Electronic Supplementary Material (ESI) for RSC Advances.
This journal is © The Royal Society of Chemistry 2018

Supporting Information

Computational Method for Estimating Progression Saturation of Analog Series

Ryo Kunimoto,[#] Tomoyuki Miyao,[#] and Jürgen Bajorath[*]

Department of Life Science Informatics, B-IT, LIMES Program Unit Chemical Biology and Medicinal Chemistry, Dahlmannstr. 2, Rheinische Friedrich-Wilhelms-Universität, D-53113 Bonn, Germany.

[#]The contributions of these authors should be considered equal.

Supplementary Table S1

Table S1. Analog series.

<table>
<tr><th>Scaffold</th><th>AID</th><th>Target name</th><th>Assayed compound CID list</th></tr>
<tr><td>[R1]C(=O)CSc1nnc(n1c2ccc(cc2)F)Cc3cccn3C</td><td>485341</td><td>Beta-lactamase</td><td>5309713|5309573|5308841|5309730|5309409|5308162|3236178|5309704|5308193|5308021|5308811|5307841|5308992|5309579|5307849|5309876|5309240|5309173|5308639|5307438|5309338|5307770|5308646|5307443|5308984|5309404|5308367|3245868|5308019|5308008|5308200|3239707|5308194|5308822|5309165|5309064|3244487|5309865|5307846|5307603|5307604|5308668</td></tr>
<tr><td>[R1]C(=O)C1CCCN(C1)S(=O)(=O)c2cccc3c2nsn3</td><td>686978</td><td>Tyrosyl-DNA phosphodiesterase 1</td><td>9551241|3242612|16020140|16020152|3242967|3235664|3238706|16020137|17588880|16020128|16000006|9550281|15995816|3243654|16020113|16001304|3235633|16001305|2813340|16020131|6619603|3244329|3241185|15994844|16002895|3238029|16002716|15997305|16020082|16020116|15999698|20855732|3240186|16020084|3245391|16001902|6621933|9551219</td></tr>
<tr><td>[R1]OCC(=O)N(CC(C)C)c1c(n(c(=O)[nH]c1=O)Cc2ccccc2)N</td><td>686978</td><td>Tyrosyl-DNA phosphodiesterase 1</td><td>16367558|24982624|24687426|24819798|24980705|24981307|24687897|24981476|24979540|24980189|24980571|24983045|24980792|24816708|24687534|24980262|24687479|24687932|39903029|24687272|24983183|24687195|24687885|24818043|24687736|24978863|24980838|24980382|24980784|24983179|24981939|24819811|24687765|24687362|24687760|7571571|24688013|24982750|24980195</td></tr>
<tr><td>[R1]C(=O)CS(=O)(=O)Cc1c(oc(n1)c2cccc(c2)OC)C</td><td>686978</td><td>Tyrosyl-DNA phosphodiesterase 1</td><td>5307092|16008061|20860781|5308309|5309818|20860727|20860783|5307631|3236024|20860750|5308507|5309531|5309848|20860742|16008083|20860724|20860779|20860723|16008079|6622276|5307282|20860755|16008081|20860749|20860777|16008085|3239528|20860741|16008053|3240144|20860739</td></tr>
<tr><td>[R1]C(=O)C1(CCN1C(=O)Cc2ccc(cc2)c3ccccc3)C</td><td>686978</td><td>Tyrosyl-DNA phosphodiesterase 1</td><td>46943548|49789274|46948072|46943128|49786447|49778118|49790772|49789561|49792757|49795426|49778904|49792666|46943611|46944009|49790748|49790223|49778478|49795821|49790462|49778247|49795595|46948577|49790916|46944051|49791251|46944138|49792614|49795187|49790272|49790525|49791129|46948386|49790339|49795497</td></tr>
<tr><td>[R1]C(=O)C1CCN(CC1)S(=O)(=O)c2cccc3c2nsn3</td><td>686978</td><td>Tyrosyl-DNA phosphodiesterase 1</td><td>3275565|3241070|2328744|2065900|15990608|2328745|3237990|15990871|20853499|15996304|3236745|9551232|3237437|20853496|20853498|2328743|16001880|15990873|7437620|15990614|16194383|20853488|3235981|15990881|15990611|3243077|6621654|15990876|9551233|15990616</td></tr>
<tr><td>[R1]C(=O)C1CCC(CC1)CNS(=O)(=O)c2cccc3c2nsn3</td><td>686978</td><td>Tyrosyl-DNA phosphodiesterase 1</td><td>20862426|16008284|6622321|16008337|9551308|20862343|16008307|20862418|16008339|16194480|20862414|16194457|20862394|16008301|9551310|16008303|20862374|9550221|16008309|9551312|16008341|6619612|20862406|20862411|16008343|3244460|20862417|9551311|20862379|16008345|20862324|16008331</td></tr>
</table>

<table>
<tr><td>[R1]N1CCN(CC1)C(c2ccccc2)c3ccccc3</td><td>686978</td><td>Tyrosyl-DNA phosphodiesterase 1</td><td>1186382|3192043|23724273|885644|16682549|5308036|2877335|663320|993406|2922351|902950|2809697|67533|23724216|5353532|24820040|741176|649238|3237463|2877708|3192041|644490|2910742|2877676|4741586|718736|808806|597354|16189400|661389|3860526|1358608|2324329|1482470</td></tr>
<tr><td>[R1]C(=O)c1ccc2c(c1)oc(n2)Cc3ccc(cc3)OC</td><td>686978</td><td>Tyrosyl-DNA phosphodiesterase 1</td><td>23723935|24790874|24791217|24792948|24819459|24819576|24790209|23723980|23724021|24817039|16029812|23723995|24791749|24817377|24790208|23723397|24791026|24790227|24792977|24790494|25381440|24793196|24819192|24793803|16029814|24790186|16029852|23723937|42287872|24816935|16029822</td></tr>
<tr><td>[R1]C(=O)C1CCN(CC1)c2c3c(ncn2)n4c(n3)CCCCC4</td><td>686978</td><td>Tyrosyl-DNA phosphodiesterase 1</td><td>20912459|24792110|20912508|16021503|16021639|16021505|20912577|16021643|20912608|16034585|16021517|16021817|20912501|16021543|16021539|20912572|16021581|16021819|16021537|16021815|20912571|20912439|20912445|16021509|16021571|16021511|20912435|16021545|16021535|16021577|16021599|16021513|16021575|16021239|20912446|16021579</td></tr>
<tr><td>[R1]C(=O)c1ccc2c(c1)S(=O)(=O)c3ccccc3C2=O</td><td>686978</td><td>Tyrosyl-DNA phosphodiesterase 1</td><td>4845254|18129737|2085752|18104837|24747282|390891|40118|3451338|2573157|7742116|2335385|2335386|2331605|16196088|24688033|24688097|16196374|2374909|18106605|2124414|4848676|9245722|3389416|2512099|7732724|24677546|4851674|2335416|2443473|24747281|4294847|2419771|8874377|2310768</td></tr>
<tr><td>[R1]C(=O)c1cc(ccc1N2CCOCC2)S(=O)(=O)N3CCCCC3</td><td>686978</td><td>Tyrosyl-DNA phosphodiesterase 1</td><td>16250058|3386140|16314658|16314014|2116743|4240912|2433199|4801278|2123938|24686097|2093009|2117968|16578765|24819714|24978907|2998561|16290344|4794726|24980211|24980872|4291700|3689276|16543799|4851275|2641100|24979527|3452399|16279501|16543802|3916428|9503329|24979571|2998382|4849374|24818082|24686348|4822263|2475489|5151913|4834367|25344859|2117883|4849818|2118407</td></tr>
<tr><td>[R1]N(C)CC1C(CN(S(=O)(=O)c2ccc(cc2O1)Br)C(C)CO)C</td><td>686978</td><td>Tyrosyl-DNA phosphodiesterase 1</td><td>46903710|46904098|46904207|46903314|46904221|46903354|46903708|46904090|46903707|46904200|46904129|46904101|46904211|46904195|46903355|46904219|46903956|51358901|46903331|46903332|46903704|46904217|46904130|46904215|46904206|46903325|46904066|46904095|46903955|46903313|46904201|46904210|46903953|46904134|46903323|46903327|46904100|46904216|46903705|46903703|46904208|46903328|46903711|46903326|51358859|51358869|46904091</td></tr>
</table>

[R1]C(=O)c1ccc(cc1)OC2CCN(CC2)C(=O)COC	686978	Tyrosyl-DNA phosphodiesterase 1	24816956|24793823|24816958|24790117|24817650|23723862|24790703|24790828|16192278|24790592|24794254|24791057|24793417|24791661|24817411|23723865|24816851|24819188|24793913|24790802|24793942|24791331|24793436|24819466|23723983|24791843|24819310|16188431|24791761|24791234|24791212
[R1]C(=O)C1CCCN(C1)c2c3c(ncn2)n4c(n3)CCCCC4	686978	Tyrosyl-DNA phosphodiesterase 1	20912255|16021785|20912293|20912219|20912227|16021789|20912277|20912386|20912433|20912391|20912278|16021813|20912351|20912285|20912373|20912400|16034583|6625875|20912224|16021755|20912316|16021781|16021779|20912307|20912231|20912341|16021811|20912339|20912343|20912294|20912300|16021753|20912398|16021757|20912229|20912239|22334742|20912368|20912426|20912299|20912279|20912267|16034581|20912378|20912399|16021787|16021807|20912305
[R1]C(=O)c1ccc(cc1)OC2CCN(CC2)Cc3ccccn3	686978	Tyrosyl-DNA phosphodiesterase 1	24791264|16192009|24792946|16191810|16190300|24790747|24790145|24816727|24793188|24790439|16192141|24790773|24794269|24817810|16190034|24794214|24817482|24818967|24791385|24817089|24790505|16188958|44171471|24819449|24792978|16187445|24790465|24817790|24790676|24817125|16190283|24790579|24817093|24790444|24792068|16188421|16187262|16189163|24790165|24819503|24791099
[R1]C(=O)C1CCCN(C1)S(=O)(=O)c2cccc3c2nsn3	686979	Tyrosyl-DNA phosphodiesterase 1	9551241|16020128|16020140|16020135|16020152|3242967|3235664|3240096|3239375|17588880|16020137|3242612|16000006|15995816|3243654|16001304|6619603|16001305|2813340|16020131|9551220|16194390|15994844|16002895|3243220|3238029|15997305|16020082|16020116|15999698|9550281|16020084|6621933|16020058
[R1]N(C)CC1C(CN(C(=O)c2c(c3ccccc3n2C)-c4ccccc4CO1)C(C)CO)C	686979	Tyrosyl-DNA phosphodiesterase 1	46903271|46903274|46903303|46903260|46903281|46903293|46903290|46903258|46903264|46903265|46903280|46903829|46903295|46903267|46903289|46903298|46903287|46903285|46903300|46903273|46903305|46903297|46903299|46903291|46903282|46903302|46903304|46903288|46903262|46903286|46903277|46903259|46903261

<table>
<tr><td>[R1]N(C)CC1C(CN(C(=O)CCCn2c(cnn2)CO1)C(C)CO)C</td><td>686979</td><td>Tyrosyl-DNA phosphodiesterase 1</td><td>44506072|44505186|44492059|44496392|44505590|44505781|44504931|44505737|44494078|44505071|44493584|44506078|44505044|44504950|44505598|44505724|44505181|44505875|44489274|44505466|44496406|44505017|44505932|44505183|44505175|44505774|44505645|44489278|44505612|44505903|44505986|44504972|44505123|44488157|44505957|44504937|44495432|44495434|44505233|44498551|44506074|44506049|44505881|44505189|44505056|44505350|44506092|44505348|44506020|44495437|44505609|44505411|44506076|44505170|44505779|44489280|44505984|44505910|44505651|44495959|44498560|44505232|44505552|44505453|44494076|44505352|44505172|46903250|44485736</td></tr>
<tr><td>[R1]C(=O)c1ccc(cc1)OC2CCN(CC2)C(=O)CCOC</td><td>686979</td><td>Tyrosyl-DNA phosphodiesterase 1</td><td>24790172|24790451|24792065|24792063|24817383|24817584|24791397|24790730|24793016|24817813|24794212|24790573|24817401|24819396|24819478|45224140|24817393|24790437|24790646|24791988|24791111|23723555|24790430|23723529|24817381|24790520|23723788|24817754|25451404|24793112|24817432|23723353|24791066|24817108</td></tr>
<tr><td>[R1]N1CCN(CC1)C(c2ccccc2)c3ccccc3</td><td>686979</td><td>Tyrosyl-DNA phosphodiesterase 1</td><td>1186382|3192043|666380|2442391|5308036|2877335|993406|902950|2809697|67533|1485885|1289403|5353532|24820040|796302|1178067|2809754|2088777|3430107|1358608|2877708|3192041|885644|2910742|644490|2877676|4741586|1547484|808806|597354|16189400|661389|2956553|3860526|20958593|2324329|741176|1076661</td></tr>
<tr><td>[R1]C(=O)c1cc(ccc1N2CCOCC2)S(=O)(=O)N3CCCC3</td><td>686979</td><td>Tyrosyl-DNA phosphodiesterase 1</td><td>3452399|2640929|16250058|16314014|4240912|2433199|2640943|4801278|16314658|24686097|2093009|16578765|24819714|24978907|2998561|16290344|2472948|24980211|4834367|3386140|4849723|16543799|4851275|24979527|4794726|16279501|3916428|9503329|24979571|2998382|3689276|24818082|4822263|2475489|2123938|5151913|24980872|25344859|2117883|4849818|2118407</td></tr>
<tr><td>[R1]C(=O)C1c2ccccc2Oc3c1cccc3</td><td>686979</td><td>Tyrosyl-DNA phosphodiesterase 1</td><td>1221732|2970994|725576|709846|2862644|669614|6602790|670827|699666|742987|743804|2939606|1246885|1928115|3236180|1984529|2380057|977577|1932603|3151963|214169|1116305|3248763|718111|2905426|717579|1928177|1931819|659939|3137305|24748013|718106|718110</td></tr>
</table>

[R1]N(C)CC1C(CN(S(=O)(=O)c2ccc(cc2O1)Br)C(C)CO)C	686979	Tyrosyl-DNA phosphodiesterase 1	46903710|46904207|46903314|46904221|46903708|46904196|46903707|46904200|46903318|46904101|46904088|46903316|46903313|46904195|46903355|46904219|46903956|51358901|46903331|46903332|46903711|46903704|46904217|46904130|46904215|46904206|46903320|46904066|46904092|46903334|46903354|46903954|46904210|46903953|46904134|46903336|46903323|46903315|51358859|46903705|46903703|46904208|46904220|46903326|46904205|51358869|46904202
[R1]C(=O)c1c2c3ccccc3n(c2c(=O)n(n1)c4ccc(cc4)OC)C	686979	Tyrosyl-DNA phosphodiesterase 1	15990995|3245156|16020091|5307294|15990993|16020099|16193504|3235918|16020009|16020103|16020062|16020101|6621876|16020072|15990991|15990989|16020064|15991019|16020093|16020036|15991021|15991001|16020047|16020035|16020095|16020019|3236606|16020089|16020011|16020037
[R1]C(=O)C1CCCN(C1)c2c3c(ncn2)n4c(n3)CCCCC4	686979	Tyrosyl-DNA phosphodiesterase 1	20912255|16021785|20912293|20912219|16021811|16021789|20912277|20912291|20912286|20912433|20912391|16021813|20912351|20912284|20912285|16034581|16034583|6625875|16021755|20912316|16021779|20912229|20912231|20912341|20912227|20912339|20912300|20912265|20912292|16021753|20912307|15987602|22334742|20912386|20912267|16021783|20912378|20912398|16021787|20912400
[R1]C(=O)C1CCN(CC1)S(=O)(=O)c2ccc3c(c2)CC(N3C(=O)C)C	686979	Tyrosyl-DNA phosphodiesterase 1	16021461|20901895|20901945|16019496|20901879|20901920|20901941|16021460|20901908|20901932|20901927|20901877|20901880|20901942|20901921|20901936|20901925|20910594|20901919|20910601|20901890|20910600|20901926|20901884|20901882|20901892|20901897|20901923|20901928|20901873
[R1]C(=O)c1ccc(cc1)OC2CCN(CC2)Cc3ccccn3	686979	Tyrosyl-DNA phosphodiesterase 1	24791264|16192009|24792946|24792068|24817235|24791011|16190300|24790145|24816727|24793188|24790439|16192141|24790773|24817810|16190034|24794214|24817482|24818967|24791385|24817089|24790505|16188958|44171471|24819449|16187445|24790465|24817790|24790676|24817125|16190283|24790579|24817093|16191810|16188421|24792119|16189163|24790165|24819503|24792978

[R1]C(=O)C1CCCN(C1)c2c3c(ncn2)n4c(n3)CCCCC4	652104	TAR DNA-binding protein 43	20912255|16021785|20912293|16021811|16021789|20912277|20912291|20912386|20912286|20912341|20912391|20912239|20912351|20912399|16021777|20912373|20912284|20912346|6625875|20912224|20912374|16021755|20912316|16021779|20912229|20912231|16034581|20912398|20912339|20912343|20912300|24793629|20912292|16021753|16021757|20912380|20912394|16021813|15987602|20912434|20912275|20912299|20912279|20912267|6625832|16021783|20912301|20912378|20912400|20912265|16021787|24792045|16021807|20912305|20912429
[R1]C(=O)CNC(=O)c1c2ccccc2c(=O)n(n1)c3ccc(cc3)OC	504467	ATPase family glucagon like peptide 1 receptor	5073112|3427503|11957274|4229929|15994628|4229931|4229926|22421613|5155222|3423835|5073298|5073296|4229933|5073289|22421602|16193117|5073286|22421611|3242338|22421616|22421606|3427500|5073114|5073115|15992264|16000190|3427505|3239051|16193207|4229928
[R1]C(=O)c1cc(ccc1N2CCOCC2)S(=O)(=O)N3CCCCC3	504467	ATPase family glucagon like peptide 1 receptor	16250058|2640892|16314014|2116743|2433199|4801278|16304608|16314658|24686097|2117968|24819714|24978907|2998561|16290344|4794726|4834367|4291700|16304609|24978939|16300637|4851275|24979527|3452399|16279501|16543799|24979571|4849374|4545742|4852767|2472948|4822263|2475489|2123938|24980872|2118407
[R1]C(=O)C1CCCN(C1)c2c3c(ncn2)n4c(n3)CCCCC4	504467	ATPase family glucagon like peptide 1 receptor	20912255|20912341|20912275|20912227|16021789|20912222|20912434|20912386|20912286|20912433|20912351|20912373|20912400|6625875|20912224|20912316|20912294|20912231|20912323|20912398|20912339|20912277|20912300|20912265|20912292|20912380|20912394|20912429|22334742|20912368|20912426|20912279|20912267|20912301|20912378|20912399|24792045|20912305|20912239
[R1]C(=O)c1cc(nc2c1cccc2)c3ccc(cc3)OC	588342	Firefly luciferase	1173898|25163483|757621|1734583|7660311|1124656|2105177|4314187|1767747|4115109|2933408|2711802|2091261|2089464|25237306|2977981|2105146|16414808|757627|2089466|2259442|2334881|4398374|2092251|1221201|2302351|25237207|2932435|2357008|757623|3690908
[R1]C(=O)CNC(=O)c1c2ccccc2c(=O)n(n1)c3ccc(cc3)OC	588342	Firefly luciferase	5155219|4229928|5073112|3427503|11957274|4229929|15994628|4229931|4229926|22421614|22421613|5155222|5073296|3427502|3423835|5073298|22421601|4229933|4229927|5077853|5073297|22421600|22421602|5155221|22421607|5073286|22421611|3242338|22421616|3236326|22421606|3427500|5073114|5155217|5073115|15992264|16193117|4229924|16000190|3427505|3239051|16193207|5073289

[R1]C(=O)c1ccc2c(c1)oc(n2)Cc3ccc(cc3)OC	588342	Firefly luciferase	24791026|24790874|24791217|24792948|24819459|24819576|23723678|23724079|24790209|24791090|16029816|24790777|24817039|16029812|24791749|24817377|24792977|23723397|23723934|24790079|24790494|24819192|24793803|16029820|16029814|24790186|24791657|16029852|24790208|24816935|16029822
[R1]N(C)CC1C(CN(C(=O)c2cc(cnc2O1)Br)C(C)CO)C	624287	Guanine nucleotide-binding protein G(s) subunit alpha isoforms short	46903902|46903894|46903914|46904243|46903909|46904242|46903812|46903917|46904074|46904254|46904077|46903896|46904258|46904108|46903816|44490305|46903912|46903926|46904071|46904110|46904245|46903911|46904117|46904067|44496831|46903892|46903928|46903811|46904261|46903920|46904120|46904246|46904075|46903904|46904240|46903950|46903905|46904256|46904250|46904070|46903915|46903908|46904119|46903897|46904113|46904260|46904123|46904121|46904241|46903951|46904080|46903895|46904251|46903923|46903919|46903918
[R1]N1CCN(CC1)C(c2ccccc2)c3ccccc3	1461	Neuropeptide S receptor	1186382|3192043|666380|1293145|2442391|2385988|3192040|5308036|2877335|663320|993406|902950|2809697|1485885|1289403|1358608|796302|1178067|2088777|796303|1208293|649238|3237463|808806|3192041|644490|2910742|2877676|1547484|2877708|3244419|789874|597354|16189400|661389|3860526|1076661|3192042
[R1]OCC(=O)Nc1cccc(c1)S(=O)(=O)NC2=NCCCCC2	485367	ATP-dependent 6-phosphofructokinase	16549997|4036552|5997229|16550000|3901789|6177132|5204354|8913492|5042170|3525948|16549995|6048013|4664437|4410458|25162024|4377778|24401253|4603685|25163699|5104961|4262418|16549996|16549998|3982526|3381464|4178437|24819867|25162368|3933895|4591220|24817950|4279458|4258124
[R1]C(=O)C1CCN(CC1)c2c3c(ncn2)n4c(n3)CCCCC4	1479	Thyroid hormone receptor beta	16021503|16021637|16021639|16021541|16021643|16021821|16034585|16021517|16021817|16021573|16021539|16021579|16021581|16021819|16021537|16021815|16021543|16021613|16021611|16021509|16021571|16021511|16021545|16021609|16021535|16021577|16021599|16021513|16021575|16021239
[R1]C(=O)CN(c1ccc(cc1)OCC)S(=O)(=O)c2c(noc2C)C	504444	Nuclear factor erythroid 2-related factor 2	3236341|3239701|5308694|5309294|5308183|20858905|16007611|5307319|5309496|5309129|5308524|4121115|5309344|5308594|5309468|5309688|16007613|20858911|5307744|5307724|5307214|20858912|20858914|20858898|5307973|16007615|5307550|5309870|5308971|5308954

[R1]C(=O)c1cc(ccc1N2CCOCC2)S(=O)(=O)N3CCCCC3	504444	Nuclear factor erythroid 2-related factor 2	16250058|2640892|3689276|16314658|4849624|16314014|2116743|4240912|2433199|4801278|16304608|2123938|24819714|24978907|2998561|4367314|4794726|24980211|24980872|4291700|16304609|4849723|16300637|16543799|16290344|4851275|2641100|24979527|3452399|9503329|16543803|4849374|4545742|16279501|2472948|4822263|2475489|4834367|4849818
[R1]N1CCN(CC1)C(c2ccccc2)c3ccccc3	624330	Rac GTPase-activating protein 1	1186382|3192043|666380|1293145|23724273|24747020|885644|902950|2385988|16682549|3192040|2809776|1075974|663320|993406|2922351|24820040|1334635|5308036|2809697|4741586|1485885|23724216|1289403|70048|718736|2406292|23724282|1358608|796302|1178067|2809754|2088777|23724265|3430107|796303|1208293|649238|3237463|2877708|3192042|3192041|2442391|2910742|644490|2877335|2877676|6420019|23724260|1547484|808806|3244419|789874|597354|16189400|661389|2956553|3860526|20958593|2324329|1076661|1482470
[R1]N1CCN(CC1)Cc2ccc3c(c2)OCO3	624330	Rac GTPase-activating protein 1	1965646|23724251|670803|773148|24746817|2838447|2905103|16189449|897932|3157432|16195060|16192957|94426|791274|16192596|1245692|783485|670283|1245914|671069|791209|201764|704380|763967|2811112|670796|9549344|714646|2811109|791168|16189425|862174|198798|2992046|24761117|2811605|8129803|3038520|3192010|896230|2454274|3290710|16241437|5308392|3820477|1137490
[R1]C(=O)c1ccc2c(c1)oc(n2)Cc3ccc(cc3)OC	624202	Breast cancer type 1 susceptibility protein	23723934|24790874|24791217|24792948|24819459|24819576|23723678|23724079|24790209|24791090|24790777|24791026|16029812|23723995|24791749|24790227|24790208|24817039|23723397|23723935|24790079|24792977|24790494|25381440|24819192|24790186|24791657|23723937|42287872|24816935
[R1]OCC(=O)N(CCOC)c1c(n(c(=O)[nH]c1=O)Cc2ccccc2)N	588590	DNA polymerase iota	24687518|24980062|24980548|24982478|24979030|24687740|24819933|24979234|24688044|24983225|24980570|24688003|24818139|24982452|24981984|16347968|24979767|24978636|24688060|24979371|16285149|24979642|24818118|24982462|16276755|24980495|24980523|24979394|24687716|8588737|24687707|24978823|24982795|24818138|24856281|18277682|24687686|24687913|25102503|24980528|24981157|24979337|16369894|24819929|24982055|24981714|25102411|24816732|24980336|24982494|24979142|24982059|25102363|24982955

[R1]Nc1cc(cc2c1ncn2C(C)C)OC(CC)CO	624296	Geminin	44143062|44143101|44143066|44143077|44143094|44143106|44143095|44143093|44143075|44143108|44143060|44143100|44143107|44143085|44143096|44143065|44143070|44143047|44143061|44143064|44143099|44143063|44143053|44143078|44143052|44143102|44143097|44143111|44143112|44143087|44143086
[R1]OCC(=O)Nc1cccc(c1)S(=O)(=O)NC2=NCCCCC2	720504	Serine/threonine-protein kinase PLK1	16549997|4036552|5997229|16550000|4968895|3901789|6177132|5204354|8913492|5042170|24686124|3525948|25163118|16549995|6048013|4410458|25162024|4377778|4262418|24401253|4603685|25163699|5104961|3417627|16549996|16549998|3982526|3381464|4178437|24819867|25162368|3933895|4591220|24817950|4279458|4258124
[R1]C(=O)c1ccc(cc1)OC2CCN(CC2)C(=O)CCOC	720504	Serine/threonine-protein kinase PLK1	24790172|24790451|24792065|24792063|24817383|24817584|24790239|24791397|24791116|24791289|24790730|24793016|24819478|24794212|24790573|24817401|24819396|24817813|45224140|24817393|24790437|24790646|24816923|24791988|24791111|23723555|24790430|23723529|24817381|24790520|23723788|24817754|25451404|24793112|24817432|23723353|24791066|24791229|24817108
[R1]Nc1cc(ccc1N2CCCC2)S(=O)(=O)N3CCOCC3	720504	Serine/threonine-protein kinase PLK1	16282216|4337594|4287235|5207383|4876694|3945324|16315565|16291637|4284114|4797771|18083658|5190535|4337596|4881318|15945364|4881660|4033193|16321430|16316751|3609499|16295580|4269617|4464021|9117152|3898981|24686575|3902670|4411088|16317322|5039304|4831361|16315480|4325672
[R1]C(=O)C1CCN(CC1)S(=O)(=O)c2ccc3c(c2)CC(N3C(=O)C)C	720504	Serine/threonine-protein kinase PLK1	20901888|20901898|20910589|20901947|20901895|20910601|20901896|20901945|20901879|20901899|20901941|20901921|16021460|20901908|20901932|20901875|20901927|20901920|20901877|20901880|20901942|20910597|20901936|20901909|20901925|20910594|20901939|20901923|20910591|20901890|20910600|20901926|20910592|20901916|20901938|20901874|20901882|20901943|20901897|20901919|20901928|20901873
[R1]N1CCN(CC1)S(=O)(=O)c2ccc(cc2)OC	504333	Bromodomain adjacent to zinc finger domain protein 2B	24792336|828758|1087315|1078633|843240|717180|756017|753490|660773|858314|17376128|2878303|2456967|6470839|2881103|2595238|1360786|2456928|2955338|1147989|1340106|17366058|797560|17191310|6469828|5300804|676048|2671235|1147613|3674699|17366197
[R1]N1CCN(CC1)S(=O)(=O)c2ccc(cc2)NC(=O)C	504333	Bromodomain adjacent to zinc finger domain protein 2B	764494|1151697|1151655|1342572|4240480|1293819|653635|1506084|2815598|1041299|1506085|4122065|1257623|2997304|1341058|2902188|3497946|16192557|2834849|991658|1298827|574577|16192573|991630|1265650|2902955|2842427|1261048|9549441|1147635|2853034|1151669|1235062|2901483|1075639|3161661|1088814

<table>
<tr><td>[R1]N1CCN(CC1)C(c2ccccc2)c3ccccc3</td><td>504333</td><td>Bromodomain adjacent to zinc finger domain protein 2B</td><td>1186382|3192043|666380|23724265|23724273|24747020|885644|902950|2385988|16682549|3192040|5308036|1075974|663320|993406|2922351|24820040|2809697|4741586|1485885|1289403|70048|2406292|23724282|1358608|796302|1178067|2809754|2088777|3430107|796303|1208293|1334635|2877708|23724260|3192041|2442391|2910742|644490|2877335|2877676|6420019|718736|808806|3244419|789874|661389|2956553|3860526|20958593|3192042|1076661</td></tr>
<tr><td>[R1]N1CCN(CC1)Cc2ccc3c(c2)OCO3</td><td>504333</td><td>Bromodomain adjacent to zinc finger domain protein 2B</td><td>1965646|23724251|670803|773148|24746817|2838447|2905103|897932|3157432|16192957|94426|791274|16192596|1245692|783485|670283|1245914|671069|791209|201764|704380|763967|2811112|670796|9549344|714646|2811109|16189425|862174|198798|2992046|24761117|2811605|23581808|3192010|3290710|16241437|5308392|3820477|1137490</td></tr>
<tr><td>[R1]N1CCN(CC1)S(=O)(=O)c2ccc(cc2)C</td><td>504333</td><td>Bromodomain adjacent to zinc finger domain protein 2B</td><td>6465212|740954|2047038|1148082|2169034|2928101|2427476|269717|777589|2444361|2102315|720863|2673930|17366414|17366248|1272766|703908|6469994|720576|4784720|2822709|77620|976855|16188491|3708238|2167092|740953|787171|849899|735815|873509|717239|2929737|2545623|2106955|654762</td></tr>
<tr><td>[R1]N1CCN(CC1)S(=O)(=O)c2ccc(cc2)F</td><td>504333</td><td>Bromodomain adjacent to zinc finger domain protein 2B</td><td>1094976|17377878|9115901|6470268|2460829|1245368|2664136|2207530|655915|4410795|2902800|711091|4820506|846131|719621|2664137|975834|6469364|646142|6467380|1263051|16192571|759033|16322155|2595240|1094979|735816|717938|1151433|1094975|3238483|5300805|2902280|704436|2710694</td></tr>
<tr><td>[R1]C(=O)c1ccc(cc1)OC2CCN(CC2)Cc3ccccn3</td><td>504333</td><td>Bromodomain adjacent to zinc finger domain protein 2B</td><td>24791264|16192009|16191810|24791011|16190300|24790145|24816727|24790439|16192141|24790773|24791409|24817810|16190034|24794214|24817482|24791385|24817089|24790505|16188958|24819449|24792978|24792118|16187445|24790465|24817790|24817125|16190283|24790579|24790444|24792068|16188421|24790747|24792119|16189163|24790165|24819503|24791099|24817101</td></tr>
<tr><td>[R1]N1CCN(CC1)S(=O)(=O)c2ccc3ccccc3c2</td><td>504339</td><td>Lysine-specific demethylase 4A</td><td>7997769|2405875|3269686|8194744|2082730|2129377|2437623|2986639|2108800|1094974|2902943|3150874|2470199|18105472|4875634|2901709|2446114|1317131|2242687|6468408|3145547|1151508|973966|3890866|1151521|703957|872090|16283830|1151515|1094973|2235691|16241885|2442477|24747454|2901944</td></tr>
</table>

<table>
<tr><td></td><td></td><td></td><td></td></tr>
<tr><td>[R1]N1CCN(CC1)S(=O)(=O)c2ccc(cc2)OC</td><td>504339</td><td>Lysine-specific demethylase 4A</td><td>2556465|828758|1087315|1078633|24792336|843240|717180|756017|753490|660773|858314|17376128|2878303|897205|2456967|6470839|2881103|2595238|651944|1360786|2456928|2955338|1340106|17366058|797560|17191310|6469828|5300804|676048|2671235|1147613|1122459|3674699|17366197</td></tr>
<tr><td>[R1]N1CCN(CC1)S(=O)(=O)c2ccc(cc2)NC(=O)C</td><td>504339</td><td>Lysine-specific demethylase 4A</td><td>764494|6603065|9549441|1151655|1342572|4240480|1293819|653635|1147635|2815598|1041299|1506085|4122065|1257623|2997304|1341058|2902188|3497946|16192557|2834849|991658|1298827|574577|16192573|991630|1265650|2902955|2842427|1261048|6469424|1506084|2853034|1151669|1235062|2901483|1075639|3161661|1088814</td></tr>
<tr><td>[R1]N1CCN(CC1)C(c2ccccc2)c3ccccc3</td><td>504339</td><td>Lysine-specific demethylase 4A</td><td>1186382|3192043|666380|23724265|23724273|24747020|885644|902950|2385988|16682549|3192040|2809776|1075974|663320|2922351|24820040|5308036|2809697|4741586|1485885|23724216|1289403|70048|718736|2406292|23724282|1358608|796302|1178067|2809754|2088777|23724260|3430107|741176|796303|1208293|649238|1334635|2877708|3192042|3192041|2442391|2910742|644490|2877335|2877676|6420019|1547484|808806|3244419|789874|597354|16189400|661389|2956553|3860526|20958593|2324329|1076661|1482470</td></tr>
<tr><td>[R1]N1CCN(CC1)Cc2ccc3c(c2)OCO3</td><td>504339</td><td>Lysine-specific demethylase 4A</td><td>24762036|1965646|23724251|670803|773148|24746817|2838447|2905103|16189449|897932|3157432|16195060|16192957|94426|100984|16192596|1245692|783485|670283|1245914|671069|791209|201764|704380|763967|841695|2811112|670796|9549344|714646|2811109|791168|16189425|862174|198798|2992046|791274|24761117|2811605|8129803|3038520|3192010|896230|2454274|3290710|16241437|5308392|3820477|1137490</td></tr>
<tr><td>[R1]N1CCN(CC1)S(=O)(=O)c2ccc(cc2)C</td><td>504339</td><td>Lysine-specific demethylase 4A</td><td>6465212|740954|2047038|1148082|2169034|7535246|2427468|2928101|2427476|269717|777589|2444361|2102315|720863|2673930|17366414|17366248|1272766|703908|6469994|720576|4784720|2822709|77620|976855|16188491|3708238|764400|740953|787171|849899|735815|856931|873509|717239|2929737|2545623|2106955|654762</td></tr>
</table>

[R1]C(=O)c1ccc(cc1)OC2CCN(CC2)Cc3ccccn3	504339	Lysine-specific demethylase 4A	24791264|16192009|24792946|24792068|24817235|24791011|16190300|24790747|24817790|24790145|24790505|24790439|16192141|24790773|24791409|24794269|24816727|24791294|24794214|24817482|24818967|24791385|24817089|24817810|16188958|24819449|24792978|16187445|24790465|24790488|24790676|24817125|16190283|24790579|24790444|16191810|16188421|16187262|24792119|16190034|24819503|24791099|24817101
[R1]C(=O)c1cc(ccc1N2CCOCC2)S(=O)(=O)N3CCCCC3	624417	Glucagon-like peptide 1 receptor	4849624|3452399|2640929|3386140|2640892|3689276|16314658|16250058|16314014|2116743|4240912|4849723|2433199|2640916|4801278|2123938|24686097|2093009|16578765|24819714|24978907|2998561|2472948|24980211|4834367|4291700|16304609|24978939|16300637|16543799|4851275|24979527|4794726|3916428|9503329|24979571|2998382|4849374|4545742|4852767|4440644|4822263|2475489|24980872|2118407
[R1]C(=O)C1CCCN(C1)c2c3c(ncn2)n4c(n3)CCCCC4	624417	Glucagon-like peptide 1 receptor	20912255|16021785|20912275|20912219|16021811|20912343|20912434|20912286|20912391|20912278|20912351|20912285|16021777|16034583|20912265|6625875|16021755|16021781|16021779|20912229|20912339|20912294|24793629|16021753|16021757|20912380|20912394|15987602|20912299|20912373|20912386|16021783|20912378|20912399|16021787|24792045|16021807|20912305|20912429
[R1]N1CCN(CC1)C(c2ccccc2)c3ccccc3	2551	Nuclear receptor ROR-gamma	1186382|3192043|666380|1293145|23724273|24747020|885644|902950|2385988|16682549|3192040|2809776|1075974|663320|993406|661389|24820040|1334635|5308036|2809697|6726|1485885|1289403|70048|1547484|2406292|23724282|1358608|796302|1178067|2809754|2088777|3430107|796303|1208293|649238|3237463|2877708|23724260|3192041|644490|2910742|718736|2877335|2877676|4741586|5353532|808806|3244419|789874|597354|16189400|2922351|2956553|3860526|20958593|3192042|1482470
[R1]C(=O)COC(=O)c1cc(ccc1N2CCOCC2)[N+](=O)[O-]	624327	Fatty acid synthase	16959066|16959065|16542150|16542089|16959024|16959044|16959042|16542101|16280467|16542151|7404270|16542091|24982449|25162050|7404267|24817951|24982398|24819998|7404282|24687427|24687991|24818124|9111282|16959059|16959045|16542152|16542086|7404239|24818143|24981005|24687295|24980216
[R1]C(=O)COC(=O)c1cc(ccc1N2CCOCC2)[N+](=O)[O-]	624326	Fatty acid synthase	16959066|16959065|16542150|16542089|16959024|16959044|16959042|16542101|16280467|16542151|7404270|16542091|24982449|25162050|7404267|24817951|24982398|24819998|7404282|24687427|24687991|24818124|9111282|16959059|16959045|16542152|16542086|7404239|24818143|24981005|24687295|24980216

<table>
<tr><td>[R1]C(=O)CS(=O)(=O)Cc1c(oc(n1)c2cccc(c2)OC)C</td><td>485364</td><td>Thioredoxin glutathione reductase</td><td>5307092|16008061|20860729|20860781|5307967|20860716|20860739|5307631|5308176|20860750|5308507|5309531|5309848|20860742|16008083|20860724|20860779|9550783|16008079|6622276|5307282|6622245|20860748|16008081|16008059|16008085|3240144|16008055|20860719|20860727</td></tr>
<tr><td>[R1]OCC(=O)N(CC(C)C)c1c(n(c(=O)[nH]c1=O)Cc2ccccc2)N</td><td>504332</td><td>Euchromatic histone-lysine N-methyltransferase 2</td><td>24980189|16367558|24982624|24687426|24980705|25162684|24981307|24687932|24979540|24687940|24980792|24687885|24687534|24980262|24687479|24980207|24983179|24980890|24687195|24983183|24980295|24818043|24687272|24978863|25162355|24687990|24980382|24980784|24981939|24819811|24687765|24687362|7571571|24688013|24982750|24982008|24980195</td></tr>
<tr><td>[R1]N1CCN(CC1)S(=O)(=O)c2ccc(cc2)NC(=O)C</td><td>504332</td><td>Euchromatic histone-lysine N-methyltransferase 2</td><td>1342572|1151697|1151655|764494|4240480|16192573|653635|1147635|1041299|1506085|4122065|1257623|1235062|2902188|3497946|16192557|991658|1298827|574577|991630|1265650|2902955|1261048|6469424|1506084|2853034|1151669|1075639|3161661|1088814</td></tr>
<tr><td>[R1]N1CCN(CC1)C(c2ccccc2)c3ccccc3</td><td>504332</td><td>Euchromatic histone-lysine N-methyltransferase 2</td><td>1186382|3192043|666380|1293145|23724273|24747020|2442391|902950|2385988|16682549|3192040|16189400|2877335|993406|24820040|1334635|6726|1485885|1289403|70048|5353532|23724282|1358608|796302|1178067|2809754|23724265|3430107|796303|1208293|649238|3237463|2877708|3192042|3192041|644490|718736|1482470|1547484|808806|789874|597354|2809776|661389|3860526|20958593|2324329|1076661|5308036</td></tr>
<tr><td>[R1]OCC(=O)N(CCOC)c1c(n(c(=O)[nH]c1=O)Cc2ccccc2)N</td><td>504332</td><td>Euchromatic histone-lysine N-methyltransferase 2</td><td>24687518|24980062|24980548|24982237|24979030|24687740|24979234|24983225|24980570|24688003|24818139|24982452|24979767|24978636|24688060|24979371|16285149|24979642|24818118|24982462|24980495|24982478|24687716|8588737|24980523|24982795|24818138|24856281|24981984|24687913|24980336|24980528|24979337|16369894|24819929|24981714|25102411|24979394|24982494|24979142|24982059</td></tr>
<tr><td>[R1]N1CCN(CC1)Cc2ccc3c(c2)OCO3</td><td>504332</td><td>Euchromatic histone-lysine N-methyltransferase 2</td><td>1965646|23724251|671069|773148|2838447|16189449|897932|16195060|16192957|94426|791274|16192596|1245692|670283|1245914|791209|201764|763967|2811112|670796|9549344|714646|198798|791168|16189425|862174|2992046|24761117|2811605|23581808|3192010|896230|2454274|3290710|16241437|5308392|1137490</td></tr>
<tr><td>[R1]N1CCN(CC1)S(=O)(=O)c2ccc(cc2)C</td><td>504332</td><td>Euchromatic histone-lysine N-methyltransferase 2</td><td>6465212|740954|2047038|1148082|2169034|2928101|2427476|777589|2444361|2102315|720863|2673930|17366248|703908|720576|4784720|17366414|77620|16188491|3708238|2167092|740953|787171|849899|735815|873509|717239|2929737|2545623|2106955|654762</td></tr>
</table>

<table>
<tr><td>[R1]C(=O)Cc1ccc(c(c1)S(=O)(=O)N2CCOCC2)OC</td><td>504332</td><td>Euchromatic histone-lysine N-methyltransferase 2</td><td>16250199|4800989|24979095|4800230|16196468|2082178|24980235|24982480|4853205|24979233|24686902|24978742|24817797|2093858|24979572|16349495|2079836|4800931|4857205|2085612|2124987|24982034|4827594|2083069|2524276|24979509|2125206|24978894|2125828|2574238</td></tr>
<tr><td>[R1]N1CCC(CC1)n2c(ccn2)NC(=O)CCCc3ccccc3</td><td>504332</td><td>Euchromatic histone-lysine N-methyltransferase 2</td><td>24817749|24790076|24790604|24817325|24790124|24791081|24790266|24793228|24790576|24790768|24790158|24790783|24793810|24790765|24791222|24817764|24791400|24793224|24794221|24793152|24794107|24791764|24790157|24819455|24818847|24791135|24817775|24790083|24790152|24790548|24792989|24819540|24791539</td></tr>
<tr><td>[R1]C(=O)c1ccc(cc1)OC2CCN(CC2)Cc3ccccn3</td><td>504332</td><td>Euchromatic histone-lysine N-methyltransferase 2</td><td>16192009|24791011|16190300|24816727|24790505|24790439|16192141|24790773|24791409|24817810|24791294|24794214|24817482|24818967|24791385|24790676|16188958|24819449|24792118|24790488|24817125|16190283|24790579|24790444|24792068|24790747|24792119|16189163|16190034|24819503|24792978</td></tr>
<tr><td>[R1]N1CCN(CC1)S(=O)(=O)c2ccc(cc2)C</td><td>540317</td><td>Chromobox protein homolog 1</td><td>6465212|740954|2047038|1148082|7535246|2427468|2928101|2427476|269717|777589|2444361|2102315|720863|2673930|17366414|17366248|1272766|703908|6469994|720576|4784720|2822709|77620|976855|16188491|2167092|740953|787171|849899|735815|856931|873509|717239|2929737|2545623|2106955|654762</td></tr>
</table>

Reported is the composition of all analog series used in our study. The core structure of each series is provided in canonical SMILES representation. AID is the PubCHEM identifier for each assay and CID the PubCHEM identifier of each assayed compound. For each assay, the target name is also provided.
